# 392. The Impact of Therapeutic Interventions on Post-Acute Sequelae of SARS-CoV-2 (PASC) in COVID-19 Survivors: A Retrospective Analysis

**DOI:** 10.1093/ofid/ofad500.462

**Published:** 2023-11-27

**Authors:** Andrea S Calderon, Kristin Englund

**Affiliations:** Cleveland Clinic, Pembroke Pines, Florida; Cleveland Clinic Foundation, Cleveland, OH

## Abstract

**Background:**

Post-acute sequelae of SARS-CoV-2 (PASC) is a condition characterized by persistent and debilitating symptoms that continue more than 28 days beyond the acute phase of COVID-19. A significant proportion of individuals who have contracted COVID-19 have reported long-term symptoms consistent with PASC. Despite the growing number of reports on PASC, its exact pathophysiology remains unclear, and there are currently no effective treatments available. Although various therapeutic interventions have been developed and used to treat COVID-19 patients, the effectiveness of these interventions in preventing the development of PASC is not yet established. Therefore, there is a pressing need to identify potential long-term health impacts and the relationship between therapeutic interventions and the development of PASC in COVID-19 survivors.

**Methods:**

A retrospective chart review was conducted at the reCOVer Clinic at Cleveland Clinic Foundation from March 2020 to November 2022. The study population consisted of patients diagnosed with PASC, who were matched with controls without PASC at a 1:1 ratio.

**Results:**

We identified 57 patients that were deemed to have PASC in the study period. Multiple interventions that were given at the time of COVID-19 diagnosis were reviewed. Table 1 shows the characteristics of both patient groups. Our analysis did not find any significant differences in the baseline characteristics of patients who did and did not develop PASC, including hospital admission status, need for ICU, and need for intubation. Among those that received nirmatrelvir-ritonavir, tocilizumab, remdesivir and dexamethasone there were no statistical differences found in those that eventually developed PASC (p-values of 0.50, 0.74, 0.07 and 0.06, respectively).

**Figure:**
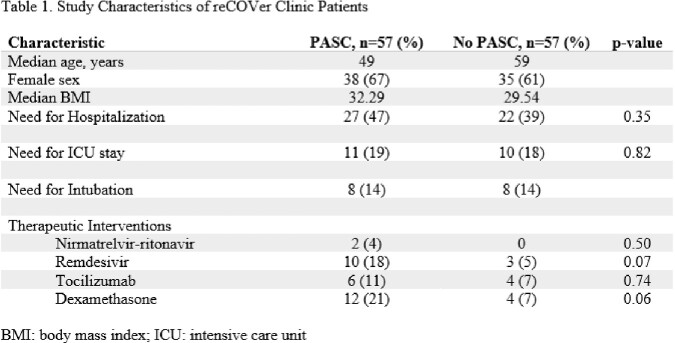

**Conclusion:**

The findings of our study suggest that treatment alone at the time of COVID-19 diagnosis is not protective against the development of PASC. Further studies are needed to identify other potential risk factors for PASC. Clinicians should consider future research focusing on identifying effective treatments for this debilitating condition.

**Disclosures:**

**All Authors**: No reported disclosures

